# The Effect of Music and Eye Masks on Sleep Quality and Delirium in Abdominal Surgery Intensive Care Patients: Randomized Controlled Trial

**DOI:** 10.1111/nicc.70072

**Published:** 2025-06-03

**Authors:** Tuğçe Topal, Yeliz Sürme

**Affiliations:** ^1^ General Surgery Intensive Care Unit Kayseri Training and Research Hospital Kayseri Türkiye; ^2^ Faculty of Health Sciences, Surgical Nursing Erciyes University Kayseri Türkiye

**Keywords:** delirium, eye masks, listening to music, nursing, quality of sleep, surgical intensive care

## Abstract

**Background:**

Postoperative delirium is a negative surgical outcome that can affect patients of any age, from children to elderly.

**Aim:**

This study evaluated the impact of eye masks and music on sleep quality and delirium among patients in surgical intensive care units.

**Study Design:**

This study was designed as a randomized controlled trial. The study sample consisted of 45 patients (21 intervention and 24 control) who were hospitalized in the General Surgery Intensive Care Unit, underwent abdominal surgery and met the inclusion criteria. Patients were assigned to the experimental and control groups by simple randomization method. Patient Identification Form, Richard‐Campbell Sleep Questionnaire (RCSQ), Nursing Delirium Screening Scale (Nu‐DESC) and Richmond Agitation‐Sedation Scale (RASS) were utilized. In the intervention group, patients were left with the eye masks applied after 22:00. Concurrently with the application of the eye masks, calming classical music was played for 1 h. Descriptive statistics, Chi‐square test, Student's *t*‐test, one‐way analysis of variance (ANOVA), two‐way repeated measures ANOVA and linear regression analysis were used. The CONSORT checklist was used in reporting the study.

**Results:**

On the third day of the study, the mean RCSQ score in the intervention group was statistically higher (72.95 ± 7.47, 49.66 ± 10.80; *p* < 0.001). The intervention group's mean Nu‐DESC score was 0.14 ± 0.35, and the control group's score was 0.83 ± 0.56 (*p* < 0.001). Additionally, throughout the study, no delirium was observed in the intervention group, while delirium developed in 8.3% of the control group. A significant positive relationship was found between the application of music and eye masks post‐surgery and the difference in RCSQ scores (*zβ*: 0.843; *p* < 0.001; [95% CI: 0.027; 0.041]). A significant negative relationship was found between the application of music and eye masks post‐surgery and the difference in Nu‐DESC delirium scale scores (*zβ*: −0.579; *p* < 0.001; [95% CI: −0.593; −0.234]).

**Conclusion:**

The research concluded that listening to calming music for 1 h daily and wearing an eye mask before sleeping increased sleep quality and decreased the incidence of delirium.

**Relevance to Clinical Practice:**

Intensive care nurses can contribute to improving patients' sleep quality and preventing delirium by applying eye masks and playing music for the patients.

**Summary**

- What is known about the topic
  ○ Insomnia is an important stressor in the intensive care unit and can cause physiological and psychological negative feedback on the immune system; insomnia can cause disruptions in melatonin levels and circadian rhythms, leading to the development of delirium.
  ○ Multifactorial non‐pharmacological interventions were effective in preventing delirium.
- What this paper adds
  ○ Although previous studies have explored the impact of earplugs and eye masks on delirium in general ICU patients, our study was conducted to reduce the rates of insomnia and delirium after abdominal surgery and is the first study in the literature in this regard.
  ○ The study revealed that music and eye masks positively contribute to the transition to sleep and prevent delirium by promoting relaxation before sleep and providing circadian rhythm in patients at risk for insomnia and delirium after abdominal surgery.
  ○ This study highlights the importance of using eye masks and music postoperatively to reduce the incidence of postoperative insomnia and delirium.

## 1 Introduction

Abdominal surgery describes surgical procedures performed on organs located in the abdominal region such as the stomach, spleen, gallbladder, liver, pancreas, small intestine and large intestine. Abdominal surgical procedures are surgical procedures in which major or minor complications may develop despite the advancement of technology and the increase in scientific evidence and recommendations [1].

After abdominal surgery, most patients experience delirium and sleeplessness because of various reasons [2, 3]. Therefore, monitoring post‐surgical high‐risk patients in the intensive care unit (ICU) is substantial to reduce the risk of perioperative morbidity [3].

Postoperative delirium (POD) is a negative surgical outcome that can affect patients of any age, from children to the elderly [4, 5]. It is stated that the main reason for POD is the inflammatory process after surgery. In particular, POD develops because of the activation of the hypothalamic–pituitary–adrenal system and subsequent extrication of sympathetic nervous system mediators such as adrenocorticotropic hormone, cortisol, vasopressin, noradrenaline, adrenaline and inflammatory mediators such as tumour necrosis factor and prostaglandin E2 [6]. Besides, metabolic irregularities, stress, pain, fluid‐electrolyte imbalance, cerebrovascular events and sleep deprivation also play a role in the development of delirium [7].

The negative consequences of POD include increased length of hospital stay, decreased mental activity, increased dementia, falls, pressure sores and increased in‐hospital mortality [6]. A meta‐analysis of surgical patients other than cardiac surgery indicated that patients with postoperative delirium experienced increased mortality rates, more frequent postoperative complications, unexpected ICU admissions and extended hospital stays compared with those without delirium [8]. Similarly, Abate et al. [9] reported the global prevalence of postoperative delirium was 20%, and the probability of mortality was approximately six times higher in patients with postoperative delirium [9].

Siddiqi et al. [10] conducted a systematic review in 2016. It showed that multifactorial non‐pharmacological interventions were effective in preventing delirium [10]. Effective non‐pharmacological treatment approaches include using an orientation board, schedule and clock; providing hydration, therapeutic activities, family member companionship, visits if companionship is not possible, increased nursing supervision, providing private rooms, music and using non‐pharmacological sleep and sleep enhancement protocols [6, 11].

Insomnia is also an important stressor in the ICU and can cause physiological and psychological negative feedback on the immune system [12]. It is also stated that insomnia can cause disruptions in melatonin levels and circadian rhythms, leading to the development of delirium [13]. The relationship between circadian rhythm, melatonin and insomnia is thought to be bidirectional. When insomnia develops, especially because of psychological and physiological factors, it can lead to circadian rhythm disruption and a decrease in melatonin secretion. For example, difficulty falling asleep because of stress can cause later exposure to light in the evening, delaying the circadian phase and suppressing melatonin secretion. This can contribute to continued sleep problems [14]. Melatonin production is suppressed when light is perceived in the environment, which can lead to decreased melatonin levels in the blood and insomnia. Melatonin supplements can help regulate the circadian rhythm and make it easier to fall asleep [15].

Noise and light are two adjustable environmental factors that contribute to insomnia in patients. In a meta‐analysis investigating the impact of eye masks and earplug use on insomnia in ICU patients, the use of earplugs and eye masks concertedly had a great impact on insomnia [16].

One of the attempts to keep the noise level low in the intensive care environment is to have patients listen to music to remove them from the ambient sound. Various mechanisms, such as relaxation, distraction and masking, have been used to clarify the effect of music on sleep [17]. A meta‐analysis conducted with intensive care patients found that both music and eye protection independently decreased insomnia, while the use of earplugs might diminish the overall benefits [18]. Another meta‐analysis shows that the use of earplugs or eye masks, separately or together, affects sleep quality in critically ill patients [19]. In the meta‐analysis conducted by Fang et al. [16], it was shown that the use of an eye mask alone significantly improved sleep quality, but the use of earplugs alone did not have a significant effect.

Numerous studies in the literature have examined the impact of earplugs and eye masks on insomnia in general ICU patients [20, 21]. A study conducted in a coronary ICU reported that using an eye mask and earplugs for three nights improved sleep quality [21]. Similarly, another study found that applying an eye mask and earplugs to coronary intensive care patients for 3 days enhanced sleep quality and reduced the severity of delirium [20].

Some patients report that earplugs cause discomfort and anxiety, so patient tolerance to earplug use is important. In addition to sedation, the use of white noise or music for auditory masking is also common [22]. Therefore, this study was completed as a randomized controlled trial to evaluate the impact of eye masks and music on sleep quality and delirium in post‐abdominal surgery patients in the ICU.

### 1.1 Research Hypotheses

- Eye masks and music have an effect on sleep quality in patients monitored in the surgical ICU after abdominal surgery.
- Eye masks and music have an effect on delirium in patients monitored in the surgical ICU following abdominal surgery.

## 2 Materials and Methods

### 2.1 Research Type

This study was designed as a randomized controlled trial. This study was registered in the clinical trial registration (https://clinicaltrials.gov/; Clinical Registration Number: NCT06268158).

### 2.2 Population and Sample

The study was completed in the General Surgery ICU of a regional hospital in the Kayseri, from March 2023 to April 2024.

The clinic has 14 beds in total and patient beds are located in single rooms. Each patient room has large windows that overlook the hospital garden and receive intense daylight. In the evenings, corridor lighting and garden lighting are reflected in the patient rooms, and patients often have sleep problems. There is no routine practice in the clinic to improve sleep quality or prevent delirium. Pharmacological methods are preferred to ensure sleep patterns in patients diagnosed with delirium in the ICU.

**FIGURE 1:**
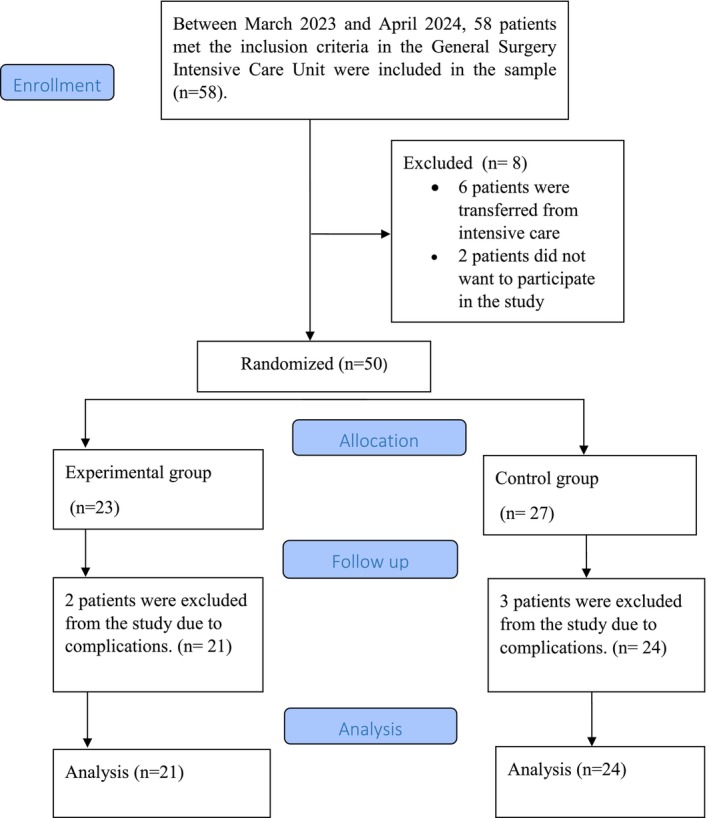
CONSORT diagram.

The study population included patients who came to the General Surgery ICU following surgery, while the sample comprised patients suitable for the inclusion criteria. G*Power 3.1.9.2 programme was utilized in calculating the sample. The sample size was planned to include a total of 34 patients, 17 in the intervention group and 17 in the control group, based on the RSC sleep scale score averages in the study by Koçak and Arslan [23] with a power of 95%, a margin of error of 0.05 and an effect size of 1.00. Considering the possible sample loss, the study planned to include 58 patients. Two patients did not accept to participate in the study, 6 patients were transferred from the ICU, and 50 patients were randomized. As a result, the study was completed with a total of 45 patients, 21 in the intervention group and 24 in the control group (Figure 1). (Supporting information).

The study inclusion criteria were as follows: (1) Patients were over 18 years of age, (2) had a Richmond Agitation‐Sedation Scale (RASS) score of −1, 0, +1, (3) received less than 2 points from the Nursing Delirium Screening Scale (Nu‐DESC) (4) had a Peripheral Oxygen Saturation (SPO_2_) of 85 and above, (5) had no communication problems (language, hearing impairment etc.) and (6) were not administered sleeping pills or sedatives.

Exclusion criteria for the study were as follows: (1) patients who underwent intervention and replacement for postoperative blood loss and fluid‐electrolyte imbalance, (2) patients with contraindications to the use of eye masks (e.g., cranial trauma), (3) patients who experienced delirium either before or after surgery, (4) patients who were intubated, (5) patients with a psychiatric diagnosis and (6) patients excluded from the study when they left the ICU early 3 nights or 4 days.

### 2.3 Randomization

With stratified randomization, researchers aim to balance the covariates between the study groups. A special stratum is created for each combination of covariates, and participants are assigned to the relevant covariate stratum. In order to assign participants to one of the groups, a simple random selection is made within each stratum [24]. A stratified randomization method was used in assigning the patients included in the study to the groups by the first researcher. The randomization method used in the study was stratified, based on age (≥ 65 vs. < 65) and sex (male/female). Within each stratum, participants were assigned to groups based on odd (intervention group *n*: 23) or even (control group *n*: 27) patient file numbers. This method was implemented as a practical assignment method instead of classical random number generation. This method is a practical stratified randomization approach that aims to provide balance between groups in terms of age and sex.

Due to the necessity of the music and eye patch application, the patients could not be blinded to the experimental groups. Since the application was carried out by the researcher working as a nurse in the same ICU, the experimental groups could not be blinded.

### 2.4 Data Collection

‘Patient Information Form’, ‘Richmond Agitation‐Sedation Scale (RASS)’, ‘Richard‐Campbell Sleep Questionnaire (RCSQ)’, and ‘Nursing Delirium Screening Scale (Nu‐DESC)’ were used in the data collection phase.

### 2.5 Patient Information Form

The form, created by the researchers based on the literature [16, 23, 25, 26], consisted of 17 questions including the patients' sociodemographic characteristics such as age, gender, education level, smoking and clinical characteristics such as whether or not they had surgery before, previous intensive care hospitalization, medical diagnosis and surgical treatment applied.

### 2.6 Richmond Agitation‐Sedation Scale (RASS)

The Richmond Agitation‐Sedation Scale (RASS) is a 10‐point system used to assess patient agitation and sedation levels. The scale score varies between +4 and −5. It includes four levels of agitation (+1 to +4, with +4 indicating a combative state), a single level for a calm and alert condition (0) and five levels of sedation (−1 to −5), with −5 representing an unarousable state. Positive RASS scores show agitated patients and negative RASS scores indicate sedated or comatose patients. It is used by health care personnel based on observation [27]. In the study conducted by Sılay and Akyol in 2018, its validity and reliability were found to be high (Cronbach's alpha coefficient 0.96) [26]. In our study, the Cronbach's alpha coefficient varied between 0.93 and 0.96.

### 2.7 Richard‐Campbell Sleep Questionnaire (RCSQ)

The RCSQ, developed by Richards in 1987, is a 6‐item scale that assesses the depth of sleep, the time taken to drop asleep, the periodicity of awakenings, the duration of alertness after awakening, sleep quality and the noise level in the environment. Each item is rated on a scale from 0 to 100. A score between ‘0 and 25’ on the scale explains very poor sleep, while a score between ‘76 and 100’ explains very good sleep. As the scale score increases, the sleep quality of the patients also increases [28]. The validity and reliability of the scale in Turkish were performed by Karaman Özlü and Özer in 2015. The Cronbach's alpha reliability coefficient was determined to be 0.91. It is based on self‐report and used by the patient [25]. In our study, the Cronbach's alpha coefficient varied between 0.85 and 0.92.

### 2.8 Nursing Delirium Screening Scale (Nu‐DESC)

The Scale was developed by Gaudreau and colleagues in 2005 specifically for nurses [29]. The scale comprises five items, including disorientation, inappropriate attitude, inappropriate communication, illusion‐hallucination and psychomotor slowing. Each item is scored between 0 and 2 points, with the total score ranging from 0 to 10. Two points and above indicate delirium [29]. The Nu‐DESC Turkish validity and reliability study was done by Çınar and Eti Aslan in 2019, and the Cronbach Alpha coefficient was reported as 0.95. It is used by health care personnel based on observation [30]. In our study, the Cronbach's alpha coefficient ranged between 0.92 and 0.96.

### 2.9 Interventions

The applications were made by the first‐line researcher, who is also an intensive care nurse. Before starting the application, a preliminary application was carried out with five patients in order to evaluate the functionality of the data collection forms, the use of eye masks and the suitability of music for the patients. The patients included in the preliminary application were not included in the experimental or control group. After the preliminary application, positive feedback was received from the patients in terms of music type and eye patch use. The comprehensibility and applicability of the data collection forms were evaluated, and no changes were made.

### 2.10 Intervention Group

On the first day, the patient introduction form was applied to the patients. During the 3 days of the study, the researcher applied the RCSQ to the patient at 10:00 AM and the Nu‐DESC at 10:00 PM. before the music and eye mask intervention. For three consecutive nights, starting at 10:00 PM, when clinical interventions were minimal, an eye mask was applied to the patients and kept on until they fell asleep. Simultaneously, calming classical music was played for 1 h. On the morning of the final day of the study, the Nu‐DESC and RCSQ were administered at the same times as they were on the first day of the study, and the study was finished for that patient.

### 2.11 Control Group

**FIGURE 2:**
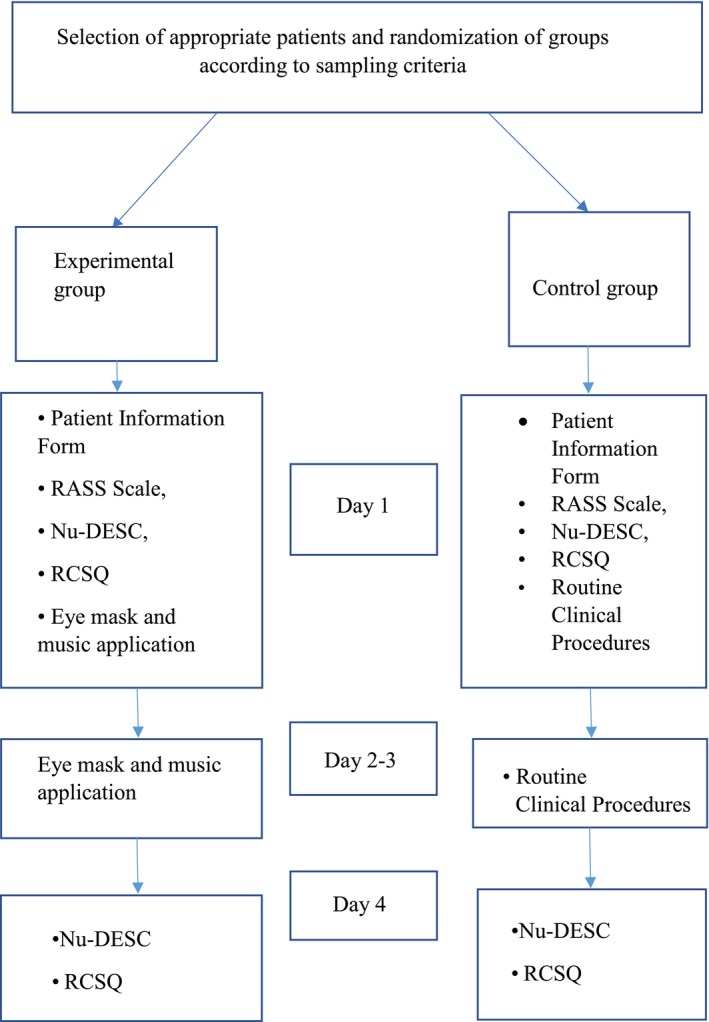
Flow chart of the study.

On the first day, the patient introduction form was applied to the patients. During the 3 days of the study, the researcher applied the RCSQ to the patient at 10:00 AM and the Nu‐DESC at 10:00 PM. Patients in the control group did not get any interventions beyond their standard clinical routines. On the morning of the final day of the study, the Nu‐DESC and RCSQ were administered at the same times as they were on the first day of the study, and the study was finished for that patient. The flow chart of the research is shown in Figure 2.

### 2.12 Materials Used

#### 2.12.1 Eye Masks

A personalized disposable eye mask that does not put any uncomfortable pressure on the eyelids and is adjusted to be comfortable without squeezing the patient's head is used to block out light.

#### 2.12.2 Music

Studies on music for sleep have used calming music that reduces muscle tension and provides relaxation [31, 32]. In the study, music was selected for its properties that facilitate falling asleep and relax (tempo of 60–80 beats/min, without accented beats). Nocturne, which means night music, has slow tempo, lyrical, sad and dreamy characteristics. Frederic Chopin's ‘Nocturne’ compositions, which have soft, gentle and calming tones, were played at a volume level that the patient could hear via a portable speaker placed in the patient's room [33].

### 2.13 Statistical Analysis

Data were examined using the IBM SPSS 25 statistical software (IBM Corp., Armonk, New York, USA). Descriptive statistics are presented as frequencies (*n*), percentages (%), means ± standard deviations (SDs) and medians with interquartile ranges (IQRs), where appropriate. The normality of numerical variables was evaluated using the Shapiro–Wilk normality test and by examining the skewness and kurtosis values. Group comparisons for categorical variables were performed using the exact method of the Pearson chi‐square test. Independent samples *t*‐test was used to compare Nu‐DESC and RCSQ mean scores between the intervention and control groups when normal distribution assumptions were met. In the comparison of the Nu‐DESC and RCSQ scores of the groups on the first, second and third days of the music and eye mask application, two‐way analysis of variance in repeated measures from general linear models was used. The predictive effect of eye masks and music on Nu‐DESC and RCSQ levels of the intervention and control groups was evaluated by linear regression analysis. In the regression model, the effect of the eye masks and music was examined by coding the intervention group (1) and the control group (0). A *p*‐value of < 0.05 was admitted for the statistical significance level.

### 2.14 Ethics Statement

Ethics Committee Permission from the XX University Clinical Research Ethics Committee (Date: 18 January 2023; Decision No: 2023/45) and institutional permission from the hospital were obtained. Written permission was gotten from individuals who agreed to participate in the study with the Informed Voluntary Consent Form.

## 3 Results

The intervention and control groups were found to be comparable in terms of age, gender, income level, education level, marital status, chronic disease status, smoking, alcohol use, whether or not they had surgery before, history of intensive care admission, diagnosis of intensive care admission, surgery performed, type of surgery, duration of stay in intensive care and duration of surgery (*p* > 0.05) (Table 1). RASS levels were also similar in the intervention and control groups throughout the research period (*p* > 0.05) (Table 2).

**TABLE 1:** Sociodemographic and clinical data of the intervention and control groups.

Sociodemographic characteristics	Intervention group		Control group		*t* (95% CI)	*p* ^a^
	$\bar{x}$ ± SD	Median (IQR)	$\bar{x}$ ± SD	Median (IQR)		
Age (years)	63.23 *±* 15.4	69.0 (53.5–72.5)	61.29 ± 13.5	63 (56.5–68)	0.449 (−6.793; 10.686)	0.656
Duration of intensive care unit stay	5.09 ± 1.51	5 (4–6)	4.54 ± 0.97	4 (4–5)	1.476 (−0.202; 1.310)	0.147
Duration of surgery	2.80 ± 1.91	2 (1.5–3.5)	3.54 ± 2.06	3.5 (1.25–5)	−1.228 (−1.934; 0.470)	0.226

Sociodemographic characteristics	Intervention group		Control group		*χ* ^2^	*p* ^b^
	*n*	%	*n*	%		
Gender						
Female	9	52.9	8	47.1	0.432	0.511
Male	12	42.9	16	57.1		
Educational status						
Literate	6	28.6	2	8.3	7.706	0.091
Primary School	9	42.9	15	62.5		
Secondary School	4	19.0	1	4.2		
High School	2	9.5	3	12.5		
University	0	0.0	3	12.5		
Marital status						
Married	17	81.0	21	87.5	0.366	0.689
Single	4	19.0	3	12.5		
Income level						
Income is less than expense	6	28.6	13	54.2	5.690	0.058
Income is equal to expense	9	42.9	3	12.5		
Income is more than expense	6	28.5	8	33.3		
Smoking						
Yes	5	23.8	7	29.2	0.164	0.685
No	16	76.2	17	70.8		
Alcohol usage						
Yes	1	4.8	1	4.2	0.009	1.000
No	20	95.2	23	95.8		
Chronic disease						
Yes	13	61.9	12	50.0	0.643	0.423
No	8	38.1	12	50.0		
Whether or not had surgery before						
Yes	16	76.2	20	83.3	0.357	0.713
No	5	23.8	4	16.7		
History of intensive care admission						
Yes	6	28.6	7	29.2	0.002	0.965
No	15	71.4	17	70.8		
Diagnosis of intensive care admission						
Trauma	2	9.5	1	4.5	0.936	0.914
Colorectal cancer	13	61.9	13	59.1		
Stomach cancer	4	19.0	6	27.3		
Pancreatic cancer	2	9.5	2	9.1		
Surgery performed						
Colorectal surgery	14	66.7	14	63.6	0.237	1.000
Gastrectomy	5	23.8	6	27.3		
Whipple surgery	2	9.5	2	9.1		
Type of surgery						
Laparoscopic	2	33.3	4	66.7	0.495	0.670
Open	19	48.7	20	51.3		
Timing of surgery						
Emergency	13	61.9	12	50.0	0.643	0.423
Elective	8	38.1	12	50.0		

^a^ An independent sample *t*‐test was performed.

^b^ Chi‐square analysis was performed.

**TABLE 2:** Comparison of postoperative RASS levels of the intervention and control groups.

RASS	Intervention group *n* (%)	Control group *n* (%)	Test value	*p* ^a^
Postoperative intensive care admission RASS				
Restless	5 (23.8%)	7 (29.2%)	0.178	0.915
Awake, calm	5 (23.8%)	5 (20.8%)		
Sleepy	11 (52.4%)	12 (50.0%)		
Day 1				
Restless	1 (4.8%)	2 (8.3%)	0.230	0.632
Awake, calm	20 (95.2%)	22 (91.7%)		
Sleepy	—	—		
Day 2				
Restless	1 (4.8%)	1 (4.2%)	0.009	0.923
Awake, calm	20 (95.2%)	23 (95.8%)		
Sleepy	—	—		
Day 3				
Restless	0 (0.00%)	6 (25.0%)	8.177	0.055
Awake, calm	21 (100.0%)	16 (66.7%)		
Sleepy	0 (0.00%)	2 (8.3%)		

^a^ Chi‐square analysis was performed.

On the 2nd and 3rd days of music and eye mask application, the mean RCSQ score was significantly higher in the intervention group compared with the control group (2nd day; 67.04 ± 7.08, 54.66 ± 10.46, respectively; 3rd day; 72.95 ± 7.47, 49.66 ± 10.80, respectively) (*p* < 0.001). It was found that the mean RCSQ score increased over time (62.76 ± 7.49; 72.95 ± 7.47) in the intervention group and reduced in the control group (*p* < 0.001) (Table 3).

**TABLE 3:** Comparison of postoperative RCSQ and Nu‐DESC score averages of the intervention and control groups.

	Intervention group		Control group		Test value	*p* ^a^
	*x̄ ±* SD	Median (IQR)	*x̄ ±* SD	Median (IQR)		
RCSQ						
Day 1	62.76 ± 7.49^A^	62 (56–67)	60.33 ± 12.94^A^	62 (50–70)	0.755 (95% CI: −4.054; 8.911)	0.454
Day 2	67.04 ± 7.08^B^	66 (62–71)	54.66 ± 10.46^B^	55 (44.5–60)	4.578 (95% CI: 6.927; 17.834)	**0.000**
Day 3	72.95 ± 7.47^C^	72 (66–80)	49.66 ± 10.80^C^	48 (42.5–54)	8.287 (95% CI: 17.619; 28.952)	**0.000**
Test statistics	*F* = 113.486; 95% CI: 2.88; 28.01; ***p*** ^b^ **0.000**		*F* = 29.602; 95% CI: 0.75; 7.40; ***p*** ^b^ **0.000**			
Nu‐DESC						
Postoperative intensive care admission	0.61 ± 0.49^A^	1 (0–1)	0.50 ± 0.51^A^	0.5 (0–1)	0.789 (95% CI: −1.185; 0.423)	0.434
Day 1	0.00 ± 0^B^	0 (0–0)	0.00 ± 0^B^	0 (0–0)	—	—
Day 2	0.047 ± 0.21^B^	0 (0–0)	0.083 ± 0.28^B^	0 (0–0)	−0.470 (95% CI: −0.189; 0.117)	0.640
Day 3	0.14 ± 0.35^B^	0 (0–0)	0.83 ± 0.56^A^	1 (0.25–1)	−4.815 (95% CI: −0.979; −0.401)	**0.000**
Test statistics	*F* = 17.941; 95% CI: 5.37; 251.03; ***p*** ^b^ **0.000**		*F* = 26.955; 95% CI: 8.14; 376.84; ***p*** ^b^ **0.000**			

^a^ An independent sample *t*‐test was performed.

^b^ Two‐way analysis of variance was performed in repeated measurements. Comparisons between times in each group, superscripts A, B and C indicate the difference between measurements.

There was no difference between the Nu‐DESC score averages of the intervention and control groups when they came to the ICU and on the 1st and 2nd days of the experiment (*p* > 0.05). On the 3rd day of the application, the Nu‐DESC score average of the intervention group was determined to be significantly lower (0.14 ± 0.35) than the control group (0.83 ± 0.56) (*p* < 0.001). (Table 3).

No delirium was noticed in either the intervention or control groups on the day of ICU admission or the 1st and 2nd days of the intervention (*p* > 0.05). On the 3rd day of the intervention, no cases of delirium were observed in the intervention group, while 8.3% of the control group developed delirium (*p* > 0.05) (Table 4).

**TABLE 4:** Comparison of intervention and control groups according to the development of postoperative delirium.

Nu‐DESC	Intervention group *n* (%)	Control group *n* (%)	Test value	*p* ^a^
Postoperative intensive care admission				
Delirium	0 (0.00%)	0 (0.00%)	—	—
No delirium	21 (100%)	24 (100%)		
Day 1				
Delirium	0 (0.00%)	0 (0.00%)	—	—
No delirium	21 (100%)	24 (100%)		
Day 2				
Delirium	0 (0.00%)	0 (0.00%)	—	—
No delirium	21 (100%)	24 (100%)		
Day 3				
Delirium	0 (0.00%)	2 (8.3%)	1.831	0.491
No delirium	21 (100.00%)	22 (91.7%)		

^a^ Chi‐square analysis was performed.

Based on linear regression analysis, there was a highly significant positive relationship between postoperative music and eye mask application and RCSQ score difference (*zβ*: 0.843; *p* < 0.001 [95% CI: 0.027; 0.041]). Additionally, 71% of the factors affecting postoperative sleep quality were explained by the music and eye mask intervention. The application of music and eye masks before sleep accounted for 71% of the improvement in sleep quality (*R* ^2^: 0.711; *F*: 105.780; *p* < 0.001).

There was a highly significant negative relationship between postoperative music and eye mask application and Nu‐DESC score difference (*zβ*: −0.579; *p* < 0.001 [95% CI: −0.593; −0.234]). 33% of the factors affecting postoperative Nu‐DESC score were explained by music and eye mask application. Pre‐sleep music and eye mask application explained 33% of the decrease in scale scores (*R* ^2^: 0.335; *F*: 21.659; *p* < 0.005) (Table 5).

**TABLE 5:** Difference between RCSQ and Nu‐DESC delirium scores at the beginning and end of the intervention: Linear regression analysis.

Regression coefficients							
	*β*	SE	*zβ*	*t*	*p*	95% confidence interval for *β*	
						Lower limit	Upper limit
RCSQ^a^							
Constant	0.498	0.041		12.153	0.000	0.416	0.581
Control (reference)							
Intervention	0.034	0.003	0.843	10.285	0.000	0.027	0.041
Nu‐DESC^b^							
Constant	0.448	0.062		7.211	0.000	0.323	0.574
Control (reference)							
Intervention	−0.414	0.089	−0.579	−4.654	0.000	−0.593	−0.234

^*^ Coded as the intervention group (1) and the control group (0).

^a^ Model statistics: *F* = 105.780; *p* = 0.000; *R* ^2^ = 0.711; adjusted *R* ^2^ = 0.704; Durbin–Watson = 1.497.

^b^ Model statistics: *F* = 21.659; *p* = 0.000; *R* ^2^ = 0.335; adjusted *R* ^2^ = 0.320; Durbin–Watson = 0.759; *zβ*: standardized regression coefficient.

## 4 Discussion

During the perioperative period, surgical stress, anaesthesia technique, hospital environment, noise and lights, night treatment and nursing checks and alarm sounds negatively affect the sleep quality of patients [34]. Postoperative sleep disturbance causes unfavourable effects on patients, including delayed healing, impairment of cognitive functions, pain sensitivity and cardiovascular issues [35]. In the study, RCSQ scores were detected to be significantly higher in the intervention group on the second and third days of the music and eye mask intervention. Moreover, linear regression analysis showed a strongly significant positive relationship between the postoperative music and eye mask application and the difference in RCSQ scores. The use of music and eye masks before sleep accounted for 71% of the improvement in sleep quality. The results of this study are consistent with the existing literature. The use of eye masks and earplugs decreases insomnia in intensive care patients, and patients sleep more [36]. Bahçecioğlu et al. [21] examined the impact of eye masks and earplugs on sleep quality, stress, fear and hemodynamic parameters of ICU patients. In their study, eye masks and earplugs were implemented in patients in the intervention group for three consecutive nights; after the intervention, heart rate, diastolic and systolic blood pressure decreased and sleep quality increased significantly [21]. It is recommended that nurses use eye masks and earplugs to increase the sleep quality of ICU patients [16, 23, 37]. In most studies involving earplugs, the physical design of the ICU is open‐plan; however, the ICUs where our study was conducted consist of single‐occupancy rooms. There is a significant difference in noise levels between single‐occupancy ICU rooms and open‐plan ICUs [38].

In this study, calming music was played to facilitate the transition to sleep in patients in single intensive care rooms. Music, which is a part of complementary treatments, is effective in improving the physiological parameters of patients by creating a relaxing effect in patients and preventing complications [39]. Since music distracts and draws attention away from pain or illness, it reduces stress and increases relaxation, thus improving sleep quality and accelerating the healing process [40]. In a systematic review, music application has been shown to have a positive impact on vital signs, sedation levels, insomnia, pain, anxiety and cosiness levels in ICU patients, and no harmful effects of music application on patients were found [41]. Kim et al. [42] determined the impact of interactive music on salivary melatonin levels, sleep quality and recovery quality in postoperative elderly patients hospitalized in the ICU. The results indicated that music intervention positively influenced sleep quality in postoperative elderly patients. However, because of the postoperative condition of the patients, no definitive conclusions could be drawn regarding the impact of the intervention on melatonin and cortisol levels [42].

The use of eye masks before sleep in the ICU environment is thought to block light, helping patients distinguish between day and night, thereby contributing to the regulation of their circadian rhythms. Additionally, playing soothing classical music before sleep is believed to reduce patients' anxiety and promote relaxation, thus facilitating the transition to sleep. The findings of this study suggest that eye masks and music can be effectively used by nurses to enhance sleep quality in patients undergoing abdominal surgery and admitted to the ICU postoperatively.

On the third day of the music and eye patch application, the mean Nu‐DESC score of the intervention group was found to be statistically significantly lower. While no one in the intervention group had delirium on the third day of the music and eye patch application, 8.3% of the control group developed delirium. A negative and highly significant relationship was found between the postoperative music and eye mask application and the Nu‐DESC score difference. Pre‐sleep music and eye mask application explain 33% of the delirium scale score decrease. The study result is parallel to the literature. Akpınar et al. [20] examined the impact of using earplugs and eye masks throughout the night on insomnia and delirium severity in ICU patients. The mean RCSQ scores of patients in the intervention group increased over time, while their mean ICU delirium screening scale scores decreased. The study concluded that the use of earplugs and eye masks during the night is associated with improved sleep quality and reduced delirium severity in ICU patients [20]. Guo et al. [43] examined the impact of non‐pharmacological interventions on postoperative delirium in elderly oral cancer patients. A programme including eye patches and music was done to the patients in the intervention group, and it was found that the frequency and duration of postoperative delirium in the patients in the intervention group decreased [43]. It has been concluded that the differentiation between day and night provided by the eye mask before sleep, along with the regulation of circadian rhythms, and the calmness and relaxation induced by music, helped patients maintain their orientation throughout their time in the ICU. As a result, this intervention prevented negative changes in consciousness and delirium.

### 4.1 Limitations

The study has several limitations. First, the sample was drawn from a single province and a general surgical ICU of one hospital. This may introduce regional bias and limit the generalizability of the findings. Another limitation is that the researcher performing the application and the patients were not blinded to the study groups. Although stratified randomization was applied according to age and gender in the study, group assignments within each stratum were made according to whether the patient file numbers were odd or even. However, because this method uses a systematic assignment method instead of true random number generation, it does not provide full statistical randomness. In this respect, it deviates from classical stratified randomization. Although this method is a practical approach, it may carry the risk of unbalanced distribution of some key variables (e.g., age, gender) among groups, especially in small sample sizes. This may reduce statistical power, prevent the effects of confounding variables from being fully controlled, limit the generalizability of the results and increase the risk of allocation bias in the assignment process. This method, while practical, may not fully eliminate the risk of allocation bias. Lastly, because the intervention duration was limited to 3 days, extending the intervention period in future research could provide clearer insights into the outcomes.

### 4.2 Implications and Recommendations for Practice

Since music and eye mask application is a complementary and alternative application without side effects, it can be integrated into intensive care nursing practices because of its contribution to the improvement in sleep quality and prevention of delirium. The use of music and eye mask should be emphasized as a complementary application in the in‐service training of intensive care nurses, and intensive care nurses should be encouraged to include these interventions in their care routines.

## 5 Conclusion

The study concluded that the use of music and the eye masks increased sleep quality and explained 71% of the increase in RCSQ scores and 33% of the decrease in delirium scale scores.

## Author Contributions

T.T. contributed to conception, data collection, design, critically revised the article, writing, editing of manuscript and final approval of the manuscript. Y.S. contributed to data analysis, review and final approval of the manuscript.

## Ethics Statement

Ethics Committee Permission (Date: 18 January 2023; Decision No: 2023/45) from the Erciyes University Clinical Research Ethics Committee and institutional permission from the hospital were obtained. This study was registered in the Clinical Trials protocol registration system (https://clinicaltrials.gov/; Clinical trial no: NCT06268158).

## Consent

Written and verbal permission was obtained from individuals who agreed to participate in the study with the Informed Voluntary Consent Form.

## Conflicts of Interest

The authors declare no conflicts of interest.

## Supporting information


**Data S1:** Supporting Information.

## Data Availability

The data that support the findings of this study are available from the corresponding author upon reasonable request.
